# High-pressure crystallization of isotactic polypropylene droplets

**DOI:** 10.1007/s00396-012-2686-y

**Published:** 2012-05-29

**Authors:** Kinga Zapala, Ewa Piorkowska, Anne Hiltner, Eric Baer

**Affiliations:** 1 Centre of Molecular and Macromolecular Studies, Polish Academy of Sciences, Sienkiewicza 112, 90 363 Lodz, Poland; 2 Center for Applied Polymer Research, Department of Macromolecular Science, Case Western Reserve University, Cleveland, OH 44106-7202 USA

**Keywords:** Isotactic polypropylene, Gamma form, High pressure, Crystallization

## Abstract

Dispersions of isotactic polypropylene (PP) particles in polystyrene (PS) were produced by interfacially driven breakup of nanolayers in multilayered systems that were fabricated by means of layer-multiplying coextrusion. The droplet size was controlled by the individual PP layer thickness ranging from 12 to 200 nm. In addition, PP was melt blended with PS to produce PP droplets larger than those formed by breakup of nanolayers. The dispersions of PP particles in the PS matrix were melted and annealed under high pressure of 200 MPa. Only the largest PP droplets, with average sizes of 170 μm, crystallized predominantly in the γ form. In the 42-μm droplets obtained by breakup of 200 nm layers, a minor content of the γ form was found whereas the smaller droplets obtained by breakup of the thinner nanolayers contained the α form and/or the mesophase. The results showed that the γ phase formed only in the droplets sufficiently large to contain the most active heterogeneities nucleating PP crystallization under atmospheric pressure. It is concluded that the presence of nucleating heterogeneities is necessary for crystallization of PP in the γ form under high pressure.

## Introduction

Isotactic polypropylene (PP) can crystallize in three crystalline forms: monoclinic α, hexagonal β, and orthorhombic γ or in the mesomorphic form. The formation of the mesophase, usually called “smectic”, requires very large undercooling, which can be reached via fast quenching. Under typical processing conditions, PP crystallizes predominantly in the monoclinic α form. The α modification exhibits lamellar branching of crystallographic origin that is unique in polymer crystallography. Such branching involves self-epitaxy on (010) crystallographic plane and leads to so-called cross-hatched morphology with a “daughter” lamellae tilted at an angle of 80° or 100° to a “mother” lamellae.

The γ modification is unusual because of a nonparallel chain arrangement. Its orthorhombic unit cell is formed by bilayers composed of parallel helices [1,2] with the direction of the chain axis in adjacent bilayers tilted at an angle of 80° to each other [1–3]. The angle between chains in adjacent bilayers is the same as between mother and daughter lamellae of the α modification. Although calculations of the packing energies of α and γ forms imply that the latter is slightly more stable [4,5], it is seldom found in samples of PP homopolymer crystallized under atmospheric pressure. The crystallization of PP in the γ form was, however, observed in the case of low molecular weight [6–9] and in the presence of chain defects or chemical heterogeneities resulted from either atacticity [10,11] or copolymerization with 1-olefin co-units [6,10,12–17]. Foresta et al. [18] demonstrated that the formation of the γ phase was enhanced by small undercooling and by nucleating agents. Crystallization of highly stereoregular iPP in the γ form is facilitated by increase of crystallization pressure [19,20]. Under elevated pressure, both the α and gamma γ phases coexist until the pressure of 200 MPa where the latter becomes dominant, although its formation is also enhanced by higher temperature. Based on their extensive experimental data, Mezghani and Phillips [20] determined equilibrium melting temperature and constructed a temperature–pressure phase diagram for the α and γ forms.

Owing to its unusual structure with nonparallel chain alignment, the γ form exhibits different mechanical properties than the α form. The plane–strain and uniaxial compression tests demonstrated higher modulus, higher yield stress and flow stress, yet slightly lower ultimate strain of γ-PP crystallized under high pressure as compared to usual α-PP [21].

The formation of the high pressure γ phase at small undercooling suggests importance of the heterogeneous nucleation. The present paper is aimed at clarifying that point by studying crystallization under high pressure in PP droplets. Polymer droplets are long known to solidify via fractionated crystallization [22–28] reflected in the presence of more than one crystallization exothermic peak. Exothermic peak at the highest temperature is attributed to crystallization from nuclei formed on the most active heterogeneities, whereas the lowest temperature exothermic peak is usually associated with homogeneous nucleation. Recently, multilayered systems of PP and atactic polystyrene (PS) were fabricated [29,30] by means of layer-multiplying coextrusion that uses forced assembly to create alternating layers of two polymers [31]. Heating films above the melting temperature of PP resulted in breakup of PP layers into droplets followed by fractionated crystallization during subsequent cooling. The droplet size, hence the crystallization behavior, was controlled by the individual PP layer thickness. Fractionated crystallization gave rise to multiple crystallization exotherms at about 40, 60, 85, and 100 °C. The exotherm at 40 °C observed for the submicron PP droplets was identified with homogeneous nucleation of the mesomorphic form. These droplets formed by breakup of the thinnest 12 nm layers were numerous enough that the majority did not contain any active heterogeneity and crystallization occurred in the form of the mesophase [29,30].

In our study, we investigated the formation of the γ phase under high pressure in PP dispersions. PP droplets with different sizes were prepared by heating PP/PS nanolayered films with various initial thicknesses of individual PP layers. In addition, melt blending of PP with PS permitted to produce PP droplets larger than those formed by breakup of the nanolayers. The PP droplets dispersed in PS were subjected to appropriate high pressure and temperature treatment to reach the region of the γ formation and stability in the phase diagram. Subsequent studies allowed to identify the crystallographic modifications formed in the droplets.

## Experimental

### Materials and samples

The studies utilized multilayered films with 257 alternating layers of PP and PS extruded on a laboratory-scale coextrusion line at Case Western Reserve University that employs layer-multiplying technology [29–31]. The isotactic polypropylene was Dow ZN5D98 with an average molecular weight *M*_w_ of about 400 kg mol^−1^ and the polydispersity *M*_w_/*M*_n_ about 5, bulk density 0.900 g cm^−3^ according to ASTM D792, and melt flow index of 3.4 g (10 min)^−1^ according to ASTM D1238. The polystyrene was Dow STYRON 685D with *M*_w_ of 527 kg mol^−1^, bulk density of 1.0450 g cm^−3^ according to ASTM D792, and melt flow index of 1.5 g (10 min)^−1^ according to ASTM D1238. The studies focused on five systems with the PP-to-PS volumetric feed ratio of 10:90, with nominal thickness of the PP layers (calculated from the composition and the film thickness) from 12 to 200 nm. These systems are referred to as PP/PS-12, PP/PS-20, PP/PS-40, PP/PS-100, and PP/PS-200, where the number holds for the nominal PP layer thickness. The detailed characterization including AFM images of these films was given in ref. [30]. Films of PS and PP were also extruded for controls. In addition, a melt blend, denoted as PP/PS-b, with PP-to-PS volumetric ratio of 30:70 was prepared by blending the components in a Brabender batch mixer at 190 °C with 60 rpm for 10 min.

## Methods

For breakup of the PP layers and also for high-pressure crystallization of PP droplets, the high-pressure cell was used as described in detail in refs. [32,33]. The cell was made of ultra high-strength steel capable of applying pressure up to 1 GPa at the temperature up to 320 °C. The samples were compressed by the use of an Instron tensile testing machine (Instron Corp., High Wycomb, UK), with velocity of the cross head of 2 mm min^−1^, via a fixture that stabilized the load exactly along the cell axis. The hydrostatic pressure inside the cell was controlled with an accuracy of ±0.5 MPa. The temperature sensor was placed 10 mm away from the sample in a narrow 1-mm thick channel, perpendicular to the wall of the cell. A temperature controller connected to four electrical heaters (600 W total power) enabled a temperature control inside the cell with accuracy of 1 °C.

To break up the PP layers into droplets, the PP/PS multilayered films assembled into packages 1.5-mm thick were placed in the high-pressure cell and subjected to pressure of 0.1 MPa, to ensure good thermal contacts of the polymer with the cell. Subsequently they were heated up to 230 °C, annealed at this temperature, and cooled to 40 °C, at which the pressure was released. Preliminary experiments demonstrated that annealing time necessary for accomplishment of PP layers breakup ranged from 3 to 30 min depending on thickness of individual PP layers. PP and PS control films were subjected to the same thermomechanical treatment.

Preliminary examination of the melt blend structure showed that PP formed elongated fibrils embedded in PS matrix. To allow formation of droplets, the blend was subjected to the same thermal treatment in the high-pressure cell as the PP/PS multilayered films, with annealing at 230 °C for 25 min.

To enable crystallization of PP in the γ form, the modified route elaborated by Lezak et al. [21,34] was applied. The samples were first pressurized to 200 MPa, then heated to 248 °C to melt the PP (at 200 MPa, the equilibrium melting temperature *T*_m_^o^ equals to 241 and 235 °C for the γ- and α form, respectively [20]) and kept at this temperature for 5 min, next cooled to 200 °C, and annealed at this temperature for 4 h. Then, the cell was cooled down to 40 °C and the pressure was released. It must be noted that at 200 MPa the glass transition temperature of PS is near 150 °C [35]. Thus, the PP droplets under the pressure of 200 MPa were surrounded by PS in the rubbery state that enabled efficient pressure transfer.

Crystallization behavior of the PP dispersions after PP layers breakup, prior to the high-pressure annealing, and also after the high-pressure annealing, was studied under atmospheric pressure using a scanning differential calorimeter, TA Instruments DSC 2920 (New Castle, DE). Specimens of the PP/PS systems and also PP and PS control specimens, with the same thermomechanical history as PP/PS, having mass of about 10 mg, were heated at 10 °C min^−1^ to 230 °C, annealed for 3 min, and cooled at 10 °C min^−1^ to room temperature under a nitrogen flow. Cooling curves of PS were normalized to the weight composition of PP/PS systems and subtracted from the thermograms of PP/PS systems to isolate the PP crystallization peaks from the superimposed glass transition of PS. In addition, PP/PS-12 specimen after high-pressure annealing was quickly heated to 90 °C, kept there for 10 min, and quenched to room temperature.

The structure of PP/PS systems, prior to and after the high-pressure annealing, was examined by scanning electron microscopy (SEM), using a JEOL JSM-5500LV (Tokyo, Japan), operating in high vacuum with accelerating voltage of 10 kV. To expose the interior of PP/PS systems, the samples were cryo-fractured. Prior to the SEM examination, the samples were sputtered with gold.

SEM technique was also utilized to measure size distributions of PP droplets in the PP/PS systems after the high-pressure treatment. Since fracture may propagate preferentially through the interface of PS and larger PP particles, to determine size distributions of particles, we followed the procedure for removal of the PS matrix developed by Masirek et al. [36]. Each specimen was dissolved in toluene to 0.6 wt% concentration. The solution was then centrifuged at 23 °C for 2 h at an acceleration of 2,859 × *g* in a centrifuge T21 Sorvall (Newton, CT). After removal of the supernatant liquid, fresh toluene was added to the sediment and the suspension was subjected to ultrasonic excitation to re-disperse the PP particles in the liquid. To remove the PS completely from the suspension, the centrifugation and subsequent re-dispersion was repeated three times. After that, the solvent was evaporated, and each specimen was sputtered with gold and examined under the SEM. In each case, diameters of about 1,000 PP particles were measured on SEM micrographs in order to determine a particle size distribution. Structure of the samples was characterized by wide-angle X-ray diffraction (WAXD) in the reflection mode. A wide-angle goniometer coupled to a PW3830 Philips (Eindhoven, The Netherlands) sealed tube X-ray generator operating at 50 kV and 30 mA was used. The X-ray beam consisted of Cu K_α_ radiation filtered by a Ni filter and electronically. The slit system that was used for collecting 2θ scans enabled collection of the diffracted beam with a divergence angle of less than 0.05°. The WAXD curve of PS was normalized to the weight composition of PP/PS systems and subtracted to isolate the PP pattern from the superimposed pattern of PS*.*

## Results and discussion

**Fig. 1:**
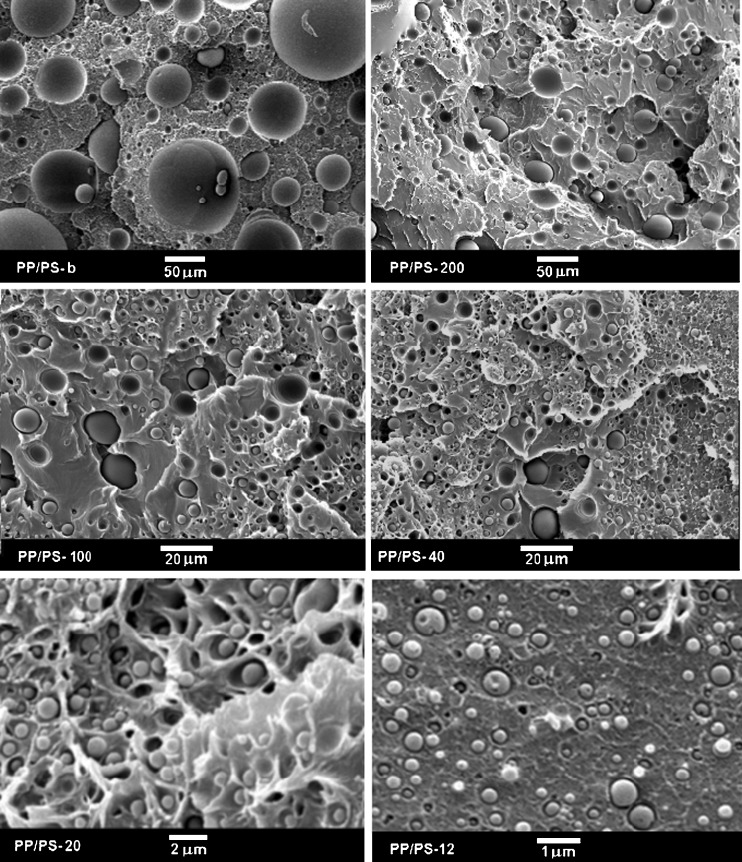
SEM micrographs of cryo-fracture surfaces of PP/PS systems, in which PP droplets formed during heating and annealing at 230 °C

SEM micrographs of cryo-fracture surfaces of PP/PS systems after the breakup of the PP layers into droplets are compared in Fig. 1. As expected, the PP layers broke into droplets of sizes increasing with increasing individual PP layer thickness as in the previous studies of Jin et al. [30]. However, even in PP/PS-200, the droplet size (diameter) did not exceed 125 μm. Larger droplets, with sizes up to 360 μm, formed only in PP/PS melt blend, which is also shown in Fig. 1.

**Fig. 2:**
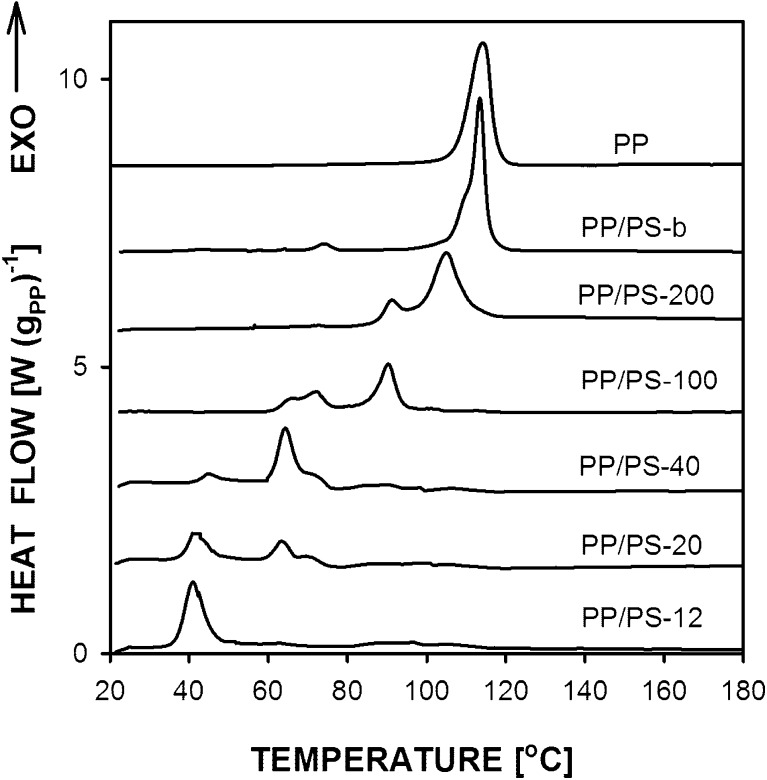
DSC cooling thermograms of PP and PP/PS with PP droplets. Prior to cooling the samples were heated to 230 °C. Heating and cooling rate 10 °C min^−1^

Figure 2 compares differential scanning calorimetry (DSC) cooling thermograms of PP and PP/PS dispersions prepared by the breakup of PP layers after subtracting the PS contribution. Only the thermogram of PP bulk is featured by a single crystallization exotherm at about 114 °C. The thermogram of melt blend PP/PS-b shows the main crystallization peak at 113 °C, with a shoulder on a descending slope, and a trace of additional peak at about 74 °C. The all other PP/PS systems exhibited pronounced fractionated crystallization with peaks at lower temperatures related to crystallization of the droplets from different nuclei as reported by Jin et al. [30]. The crystallization exotherm of PP/PS −200 was featured by two peaks centered at 105 and at 91 °C. The other PP/PS systems exhibited exotherms at about 90, 70, 65, and near 40 °C. For PP/PS-40 and PP/PS-20, the exotherm at 70 °C showed up as a shoulder on an ascending slope of the peak centered at 65 °C. In general, the temperature range of crystallization was reproducible for each material although ratios of crystallization enthalpies associated with the peaks changed to some extent. A pronounced peak at about 40 °C was observed for PP/PS-12, similarly as reported by Jin et al. [29,30], who identified this exotherm with homogeneous nucleation in the submicron PP droplets leading to formation of the mesophase, whereas the exotherms at higher temperatures seen for larger droplets were attributed to crystallization in the α form from heterogeneous nuclei.

**Fig. 3:**
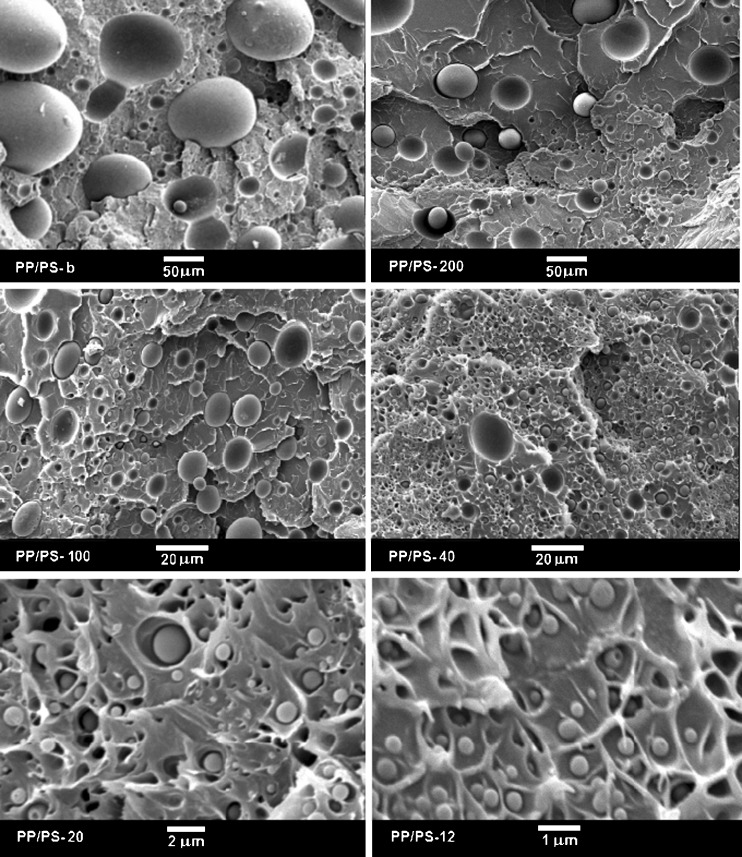
SEM micrographs of cryo-fracture surfaces of PP/PS systems with PP particles after crystallization under high pressure of 200 MPa

**Fig. 4:**
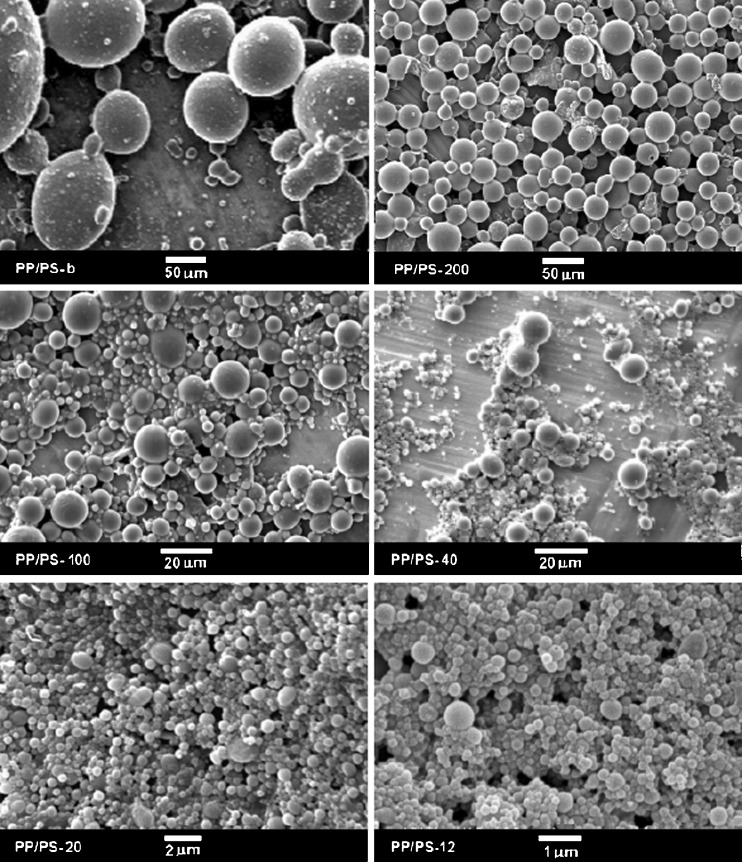
SEM micrographs of PP particles isolated from PP/PS systems after crystallization under high pressure of 200 MPa

**Fig. 5:**
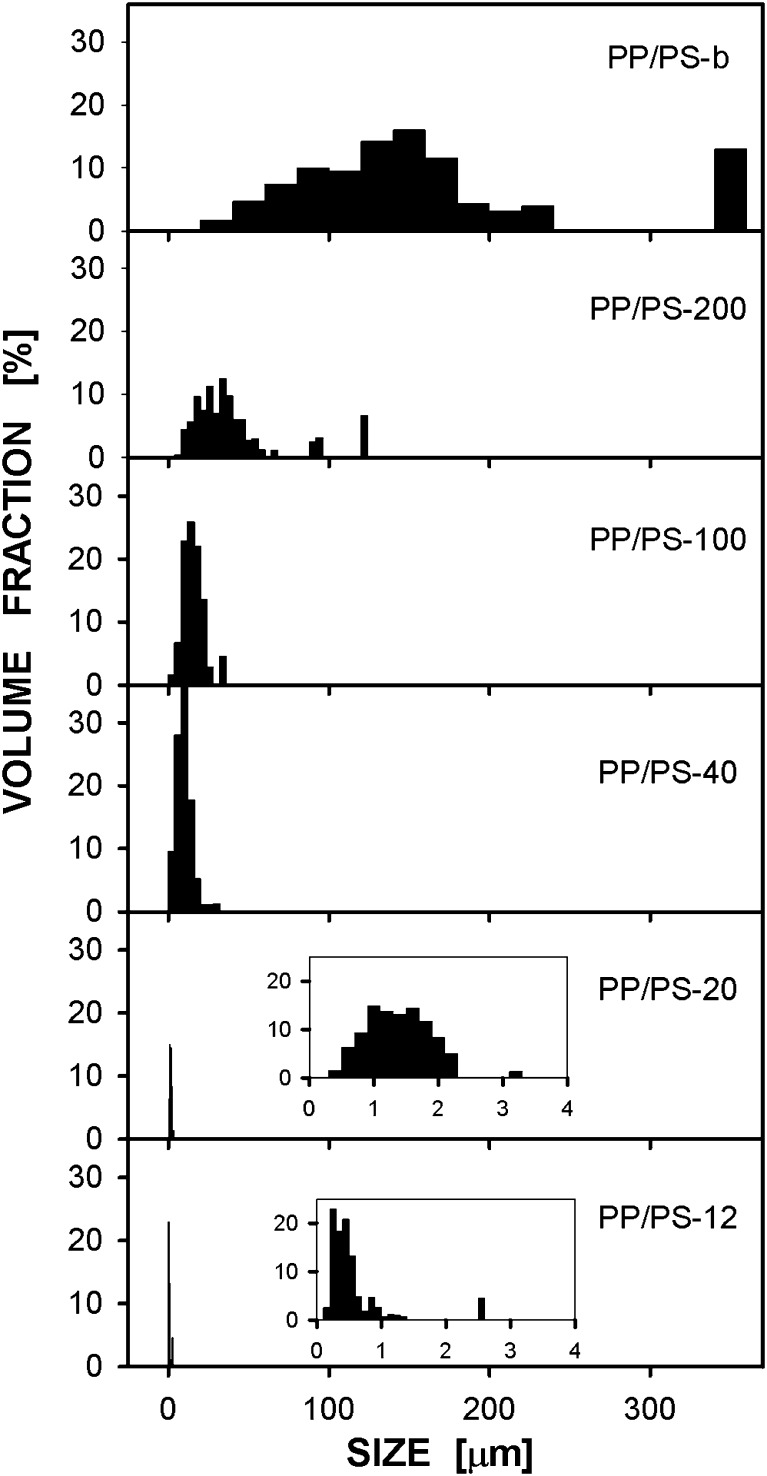
Size distributions of PP particles in PP/PS systems after high-pressure crystallization

The high-pressure treatment did not influence markedly PP droplets sizes as can be concluded from comparison of SEM micrographs in Fig. 3, showing cryo-fractured surfaces of the PP/PS specimens after high-pressure annealing, with those in Fig. 1. SEM micrographs of PP particles extracted from the high-pressure annealed specimens are collected in Fig. 4, whereas size distributions of these particles are compared in Fig. 5. A volume average droplet size was 0.6 μm for PP/PS-12, 1.5 μm for PP/PS-20, 9 μm for PP/PS-40, 20 μm for PP/PS-100, 42 μm for PP/PS-200, and 170 μm for PP/PS melt blend.

**Fig. 6:**
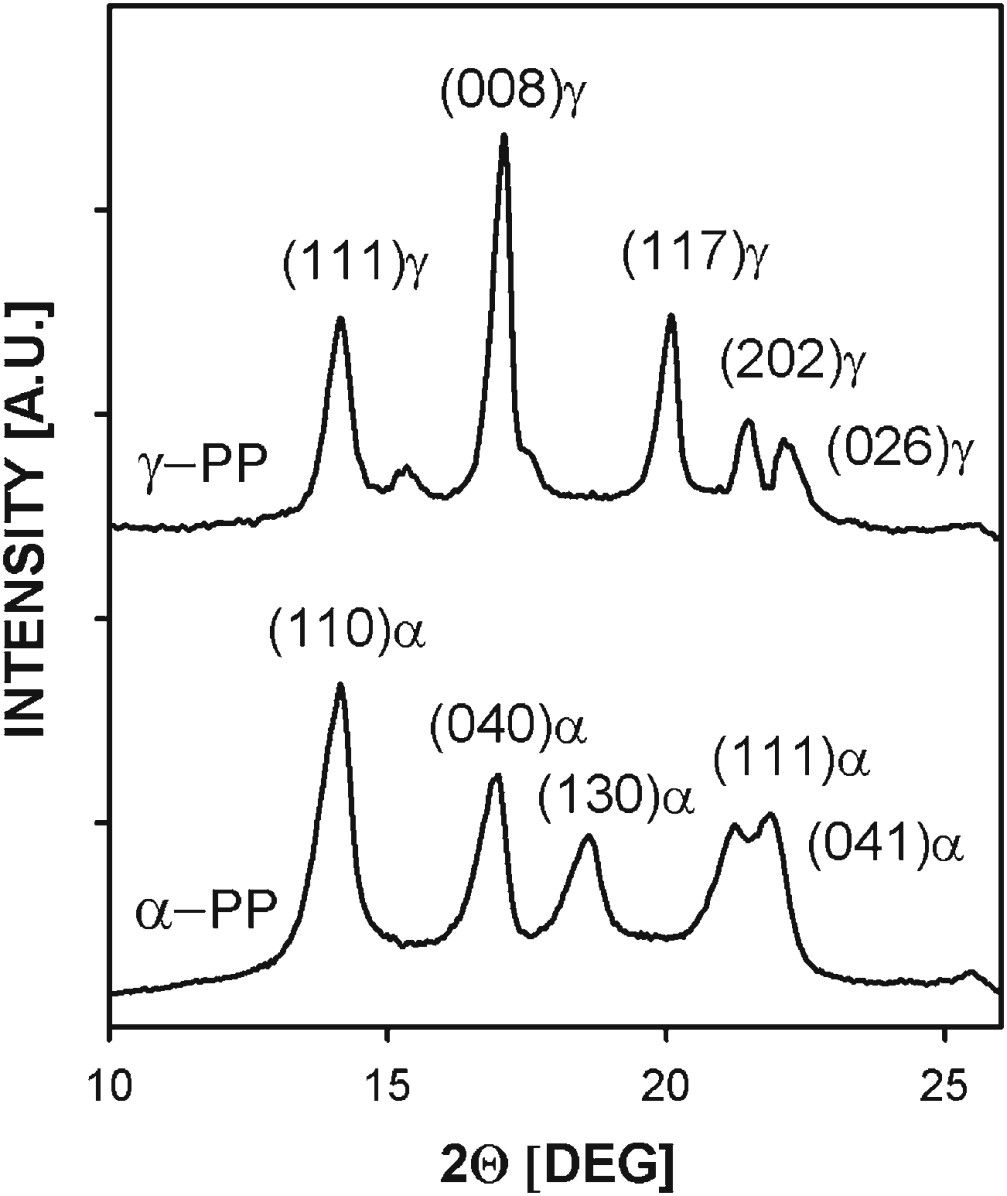
WAXD diffractograms of PP control sample crystallized in the γ form under high pressure of 200 MPa and the same sample heated and crystallized in the α form during cooling in DSC

Identification of the PP phase structure is possible via X-ray diffraction. Most of the peaks characteristic of the α and γ phases are located at nearly the same positions. Therefore, identification of the crystallographic forms has to involve analysis of diffraction curves for 2θ ranging from 18 to 21°, where two well-separated diffraction peaks of (130) plane of α crystals (2θ *=* 18.55°) and (117) plane of γ crystals (2θ *=* 20.07°) are located. According to Turner-Jones [10], the content of the γ modification, *K*_γ_, in the crystalline phase of PP sample containing both α- and γ phases can be calculated based on the following equation:

1 $$ K_{\gamma } = I_{\gamma } {\left( {117} \right)}/{\left[ {I_{\gamma } {\left( {117} \right)} + I\alpha {\left( {130} \right)}} \right]} $$

where *I*_γ_(117) and *I*_α_(130) denote integral intensities of the (117)γ and (130)α diffraction peaks, respectively. *K*_γ_ ranges from 0 to 1 for PP with the γ phase contents from 0 to 100 %. Figure 6 compares diffraction curve recorded for the PP control specimen annealed under high pressure with that for the same PP specimen subsequently melted and crystallized under atmospheric pressure. As can be seen from Fig. 6, the PP specimen annealed under the high pressure showed only the γ form, as can be concluded from the presence of (117)γ peak and absence of (130)α peak, whereas the same specimen re-melted and crystallized under atmospheric pressure during cooling in the DSC contained exclusively the α modification.

**Fig. 7:**
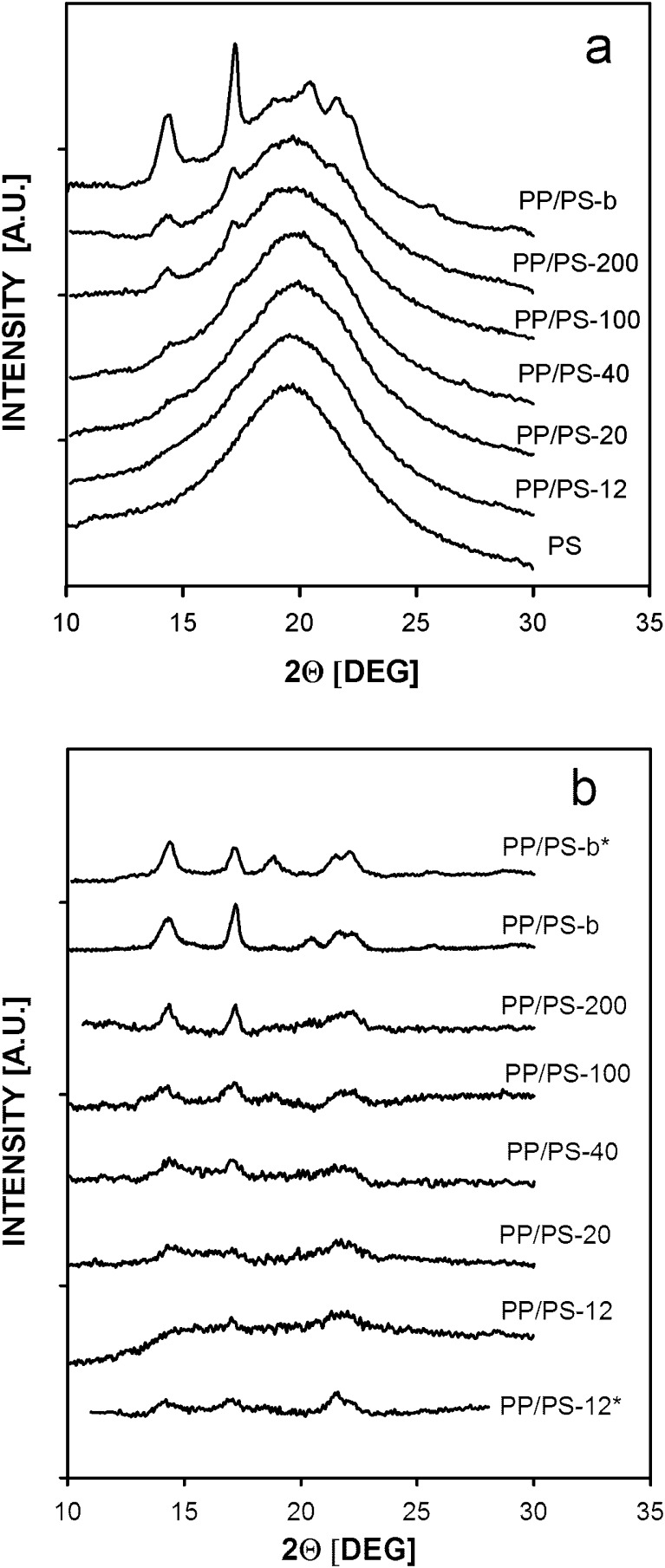
WAXD diffractograms of PP/PS systems with PP particles after crystallization under high pressure of 200 MPa: **a** before subtraction of the PS contribution and **b** after subtraction of the PS contribution. The *asterisk* denotes sample, which after high-pressure annealing underwent additional thermal treatment under atmospheric pressure: PP/PS-b* was re-melted and crystallized during cooling in DSC, PP/PS-12* was heated to 90 °C, annealed for 10 min and quenched to room temperature. *PP/PS-b* and PP/PS-b* diffractograms normalized to PP content in the other systems

**Fig. 8:**
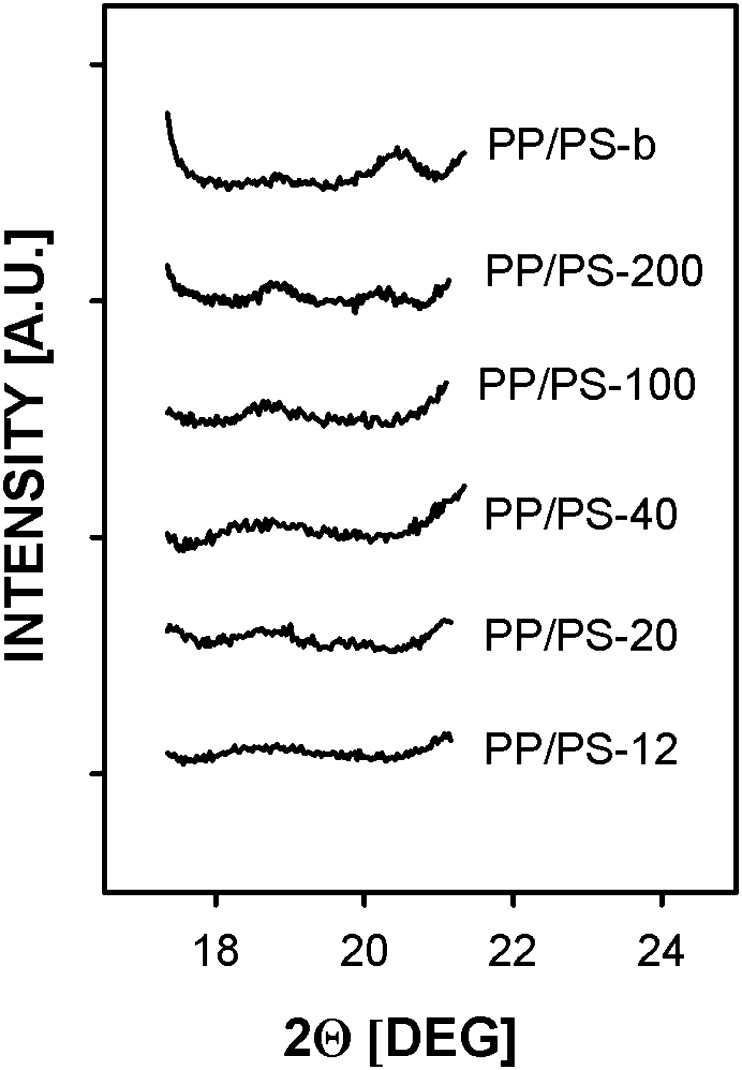
WAXD diffractograms of PP/PS systems recorded with increased acquisition time; the PS contribution subtracted, PP/PS-b diffractogram normalized to PP content in other systems

Figure 7a compares WAXD diffractograms of PP/PS systems and PS control sample after annealing under high pressure, whereas Fig. 7b displays these curves after subtracting the PS contribution. WAXD diffractograms of the PP/PS samples annealed under high pressure recorded in the 2θ range from 17 to 21° with increased acquisition time are collected in Fig. 8. The diffractograms of the melt blend PP/PS-b shown in these figures were normalized to the PP content in the other systems.

Only the diffraction curve of PP/PS-b with 170 μm PP particles, annealed under high pressure, was featured by the pronounced (117)γ peak evidencing the presence of γ phase although a small (130)α peak was also recorded for this material. A diffraction curve recorded for PP/PS-200 with 42 μm PP particles evidenced the predominant α phase with minor amount of the γ modification reflected in a small (117)γ peak. Figure 7b also shows that with a decrease of the droplet size, intensity of peaks characteristic of the α phase decreased. A diffracting curves recorded for PP/PS-12 with 0.6 μm particles after subtraction of the PS contribution showed the two broad peaks of mesomorphic PP at 15° and 21°, whereas on those of PP/PS-20 and PP/PS-40, very weak reflections of the PP α form were superimposed on the broad reflections of the mesophase, similarly as reported previously for PP dispersions crystallized under atmospheric pressure by Jin et al. [30]. The presence of the γ phase in PP/PS-b and PP/PS-200 and the absence of it in the other systems are further evidenced on Fig. 8. The content of the γ phase, estimated based on Eq. (1), was approx 90 % for PP/PS-b and approx 30 % for PP/PS-200.

Figure 6 shows that PP control sample, which crystallized under high pressure in the γ form but after that was re-melted and solidified under atmospheric pressure in the DSC, contained exclusively the α modification. Similarly, PP/PS-b, which crystallized predominantly in the γ form under high pressure, after re-melting and crystallization in the DSC contained solely the α modification, as shown by respective WAXD diffractogram in Fig. 7b. This proves that the high-pressure annealing did not cause any such change in the molecular characteristics of PP that could have increased its susceptibility to crystallization in the γ form.

In addition, Fig. 7b shows also the diffractograms of PP/PS-12 specimen which was first annealed under high pressure and after cooling underwent additional thermal treatment; it was heated to 90 °C, annealed there for 10 min, and quenched to room temperature. The diffractogram of this specimen is featured by weak peaks typical of the α form evidencing that the mesomorphic phase transformed into the α form as in the previous studies of Jin et al. [29].

**Fig. 9:**
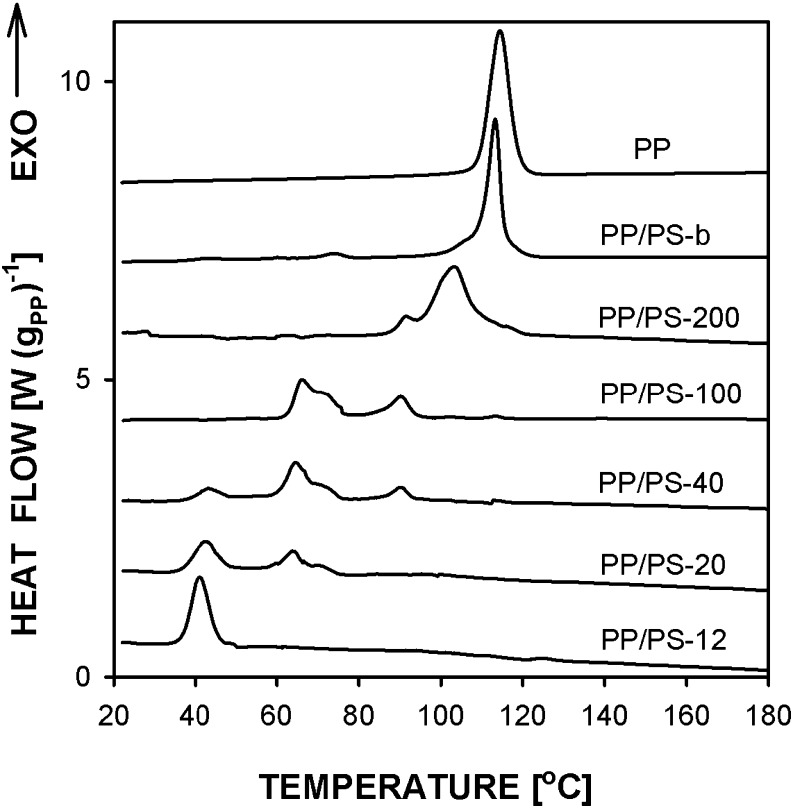
DSC cooling thermograms of PP and PP/PS systems with PP droplets crystallized under high pressure of 200 MPa. Prior to cooling the samples were heated to 230 °C. Heating and cooling rate 10 °C min^−1^

High-pressure crystallization behavior of the PP dispersions can be correlated with their crystallization under atmospheric pressure. Figure 9 compares DSC cooling thermograms of PP and PP/PS dispersions, which were annealed under high pressure and next re-melted and cooled in the DSC. The thermograms are similar to those shown in Fig. 2 indicating that the high-pressure treatment did not change temperature ranges in which the materials crystallized during cooling in the DSC. The thermograms of PP control sample shows a single crystallization peak at 114 °C, whereas that of PP/PS-b is featured by a main peak at 113 °C with low temperature shoulder and a trace of peak at 74 °C. The all other PP/PS systems exhibited fractionated crystallization as before the high-pressure annealing, with the same peak positions, with accuracy of 1.5 °C. It can be noticed that the crystallization exotherm of PP/PS-200 started to rise at similar temperature as those of PP control sample and PP/PS-b, which implies that in a significant fraction of PP droplets the nucleation was of the same nature as in these two systems.

The WAXD examination demonstrated that, besides the PP control sample, the γ phase formed predominantly only in sufficiently large PP droplets, that is in PP/PS-b with average droplet size of 170 μm and to some extent in PP/PS-200 with average droplet size of 42 μm. The PP dispersions with smaller droplets contained only the α form and/or the mesophase. We hypothesize that the γ phase crystallized in large droplets under 200 MPa during annealing at 200 °C, whereas the α phase and the mesophase formed in smaller droplets during post-annealing cooling.

Mezghani and Phillips [20] predicted the transition temperature above which the PP should crystallize exclusively in the γ form because of its lower free Gibbs energy and confirmed the predictions experimentally. However, below the transition temperature, where only the α from was expected, both forms crystallized. This was attributed to variations of tacticity, enhancing the crystallization of γ form. Under the pressure of 200 MPa, the predicted transition temperature was 174 °C. Indeed, at 176.1 °C and above, exclusively the γ crystals formed, whereas in the range from 145.5 to 164.6 °C both forms were found. Nevertheless, a region of pure α crystals in low crystallization temperature range was postulated in ref. [20], even under the pressure of 200 MPa, although no such experimental results were shown.

In our studies, both the PP control and PP/PS-b crystallized in the DSC at the highest temperature at about 113–114 °C, which indicates that the crystallization was nucleated by the most active heterogeneities. Also, a significant part of PP in PP/PS-200 crystallized at the highest temperature. In the all other systems, with smaller droplets, PP crystallization occurred at lower temperatures, being nucleated either heterogeneously on less active heterogeneities or homogeneously. Therefore, it can be concluded that the γ modification formed under high pressure of 200 MPa only in those PP droplets, which were sufficiently large to contain the most active heterogeneities able to nucleate PP crystallization in the usual α form under atmospheric pressure. Jin et al. [30] attributed the DSC crystallization exotherms of PP dispersions in the range from 60 to 90 °C to crystallization nucleated heterogeneously, although by less active heterogeneities. Under 200 MPa at 200 °C undercooling for the both forms was small, 41 °C for the γ form and 35 °C for the α form. Most probably at such small undercooling, only the most active heterogeneities were able to nucleate PP crystallization. As a result, crystallization under 200 MPa occurred in the PP dispersions with less active heterogeneities not during annealing at 200 °C but during the post-annealing cooling. The weak nucleation activity of these heterogeneities, reflected in low temperature crystallization exotherms under atmospheric pressure, allowed to reach the α form region even under 200 MPa.

The concept of epitaxial mechanism of heterogeneous nucleation requires matching between periodicities of substrate and polymer crystal structures. It was demonstrated that the α phase of PP is nucleated by two families of substrates: the first matching periodicities on the (010) face and the second matching periodicity on the (110) face [37]. The first epitaxy applies also for the γ phase. Therefore, heterogeneous nucleation of the γ modification on heterogeneities able to nucleate the α phase seems to be very probable. On the other hand, the ongrowth of the γ phase on the α phase lamellae was also reported [17,19,21,38]. Recently, Lezak et al. [21] found that under high pressure of 200 MPa the growth of γ lamellae was initiated on “seeds” consisting of a spine of single α lamella and several shorter α lamellae, although no trace of the α form was detected in ref. [21] by DSC and WAXD. Therefore, the γ phase in the PP control sample and in the large PP droplets studied by us could also grow on the α seeds nucleated by the active heterogeneities

## Conclusions

In this study, we examined crystallization of PP droplets under high pressure, in that region of the phase diagram where the γ phase is stable and formed in PP control sample, that is under high pressure of 200 MPa at 200 °C. High-pressure crystallization of PP droplets depended, however, on droplet sizes. The γ phase was found to form predominantly only in the largest droplets, with average size of 170 μm, in the melt blend, which contained the heterogeneities most actively nucleating the PP crystallization under atmospheric pressure, at the same temperature as in the PP control sample. A minor content of the γ phase was also found in the 42-μm droplets obtained by breakup of 200 nm PP layers, which under atmospheric pressure started to crystallize at the same temperature as the PP control sample and PP/PS blend. The smaller droplets obtained by breakup of the thinner layers were numerous enough that the majority did not contain those most active heterogeneities and crystallization under atmospheric pressure occurred in them mostly at lower temperature from nuclei either homogeneous or formed on less active heterogeneities. In these droplets, the γ phase did not form under high pressure. The results indicate undoubtedly that, under the conditions of our experiments, actively nucleating heterogeneities were necessary for the formation of the high-pressure γ phase of PP. It may be a general characteristic for the formation of the γ phase of PP under high pressure.
